# Evolution-informed therapy for kidney disease

**DOI:** 10.1093/emph/eoad027

**Published:** 2023-08-28

**Authors:** Robert L Chevalier

**Affiliations:** Department of Pediatrics, University of Virginia School of Medicine, Charlottesville, VA 22908, USA

## APOL1-MEDIATED KIDNEY DISEASE

A recent editorial highlighted the challenges of bridging the great divides between evolutionists and clinicians [1]. Global prevalence of chronic kidney disease is rapidly increasing and affects African Americans at 4-fold the rate for European Americans [2,3]. Social inequalities contribute to many health disparities affecting African Americans, and the discovery of G1 and G2 *APOL1* gene variants prevalent in 13% of this population contributes to the genetic component of the excess risk for nondiabetic kidney failure [4]. Focal segmental glomerulosclerosis (FSGS), the most common primary glomerular disorder causing kidney failure in the USA, is also more common in persons of African than European origin [5]. Importantly, FSGS is associated with the *APOL1* gene variants common in African chromosomes but absent in European chromosomes [6]. With the exception of SGLT2 inhibitors [7], effective therapies to slow or prevent progression of FSGS are not currently available.

## EVOLUTIONARY PERSPECTIVES

APOL1 is a serum factor that lyses trypanosomes (parasites responsible for sleeping sickness) that evolved as a host defence mechanism through natural selection in human ancestors who produced a variant of APOL1 that killed a trypanosome subspecies endemic in west Africa at the time [8]. Parasite subspecies subsequently evolved resistance proteins. This was followed by evolution of new G1 and G2 variants of human APOL1 that kill extant trypanosomes in heterozygote hosts but also increase susceptibility to podocyte injury and FSGS in hosts with two variant alleles [8]. After secretion into the circulation, APOL1 forms a complex with a host protein that is acquired by the trypanosome by endocytosis. Once incorporated in the parasite the complex is catalysed to an ion channel that promotes lysis of the trypanosome [8].

## FUTURE IMPLICATIONS

**Figure 1.**
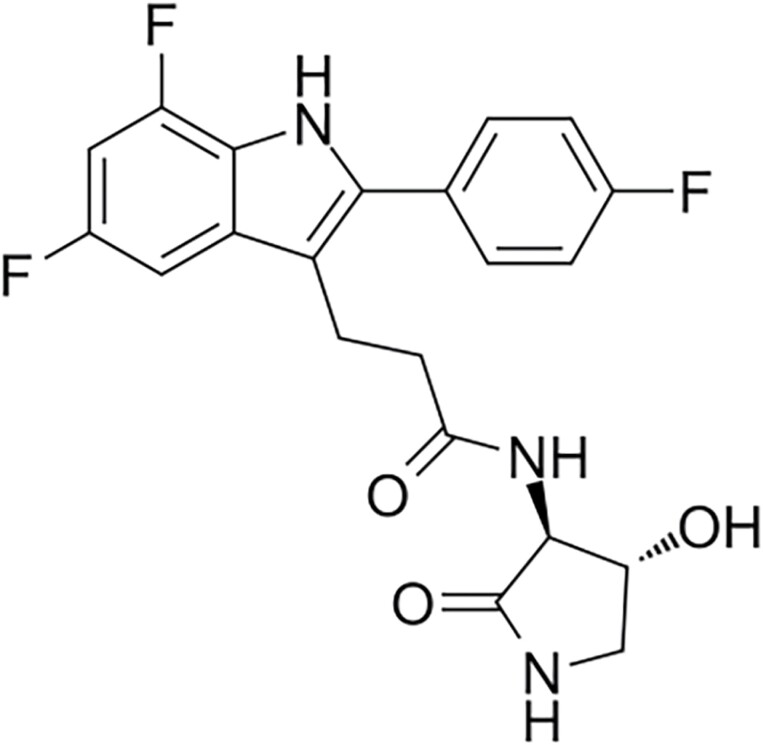
Inaxaplin.

A pharmaceutical company developed inaxaplin (Fig. 1), a small molecule that selectively inhibits APOL1 channel function in human embryonic kidney cells expressing the G1 and G2 variants [9, 10]. Moreover, treatment with inaxaplin of an APOL1 G2-homologous transgenic mouse resulted in reduced proteinuria.

In phase 2a clinical study of patients with proteinuric FSGS, treatment with inaxaplin resulted in a 48% reduction in proteinuria [9]. If confirmed in larger clinical trials (now underway), this application of molecular technology to address a major health disparity may contribute to greater awareness of the value of an evolutionary perspective in confronting public health challenges [1, 11].

## AUTHOR CONTRIBUTIONS

Robert L. Chevalier (Conceptualization [ideas; formulation or evolution of overarching research goals and aims], Investigation [conducting a research and investigation process, specifically performing the experiments, or data/evidence collection], Project Administration [management and coordination responsibility for the research activity planning and execution], Resources [provision of study materials, reagents, materials, patients, laboratory samples, animals, instrumentation, computing resources, or other analysis tools], Supervision [oversight and leadership responsibility for the research activity planning and execution, including mentorship external to the core team], Validation [verification, whether as a part of the activity or separate, of the overall replication/reproducibility of results/experiments and other research outputs], Visualization [preparation, creation and/or presentation of the published work, specifically visualization/data presentation], Writing – Original Draft Preparation [creation and/or presentation of the published work, specifically writing the initial draft (including substantive translation)], Writing – Review & Editing).
